# Group and Shuffle Convolutional Neural Networks with Pyramid Pooling Module for Automated Pterygium Segmentation

**DOI:** 10.3390/diagnostics11061104

**Published:** 2021-06-17

**Authors:** Siti Raihanah Abdani, Mohd Asyraf Zulkifley, Nuraisyah Hani Zulkifley

**Affiliations:** 1 Department of Electrical, Electronic and Systems Engineering, Faculty of Engineering and Built Environment, Universiti Kebangsaan Malaysia, Bangi 43600, Malaysia; raihanah.abdani@siswa.ukm.edu.my; 2 Community Health Department, Faculty of Medicine and Health Sciences, Universiti Putra Malaysia, Serdang 43400, Malaysia; GS52834@student.upm.edu.my

**Keywords:** pterygium assessment, group convolution, spatial pyramid pooling module, semantic segmentation, shuffle convolution

## Abstract

Pterygium is an eye condition that is prevalent among workers that are frequently exposed to sunlight radiation. However, most of them are not aware of this condition, which motivates many volunteers to set up health awareness booths to give them free health screening. As a result, a screening tool that can be operated on various platforms is needed to support the automated pterygium assessment. One of the crucial functions of this assessment is to extract the infected regions, which directly correlates with the severity levels. Hence, Group-PPM-Net is proposed by integrating a spatial pyramid pooling module (PPM) and group convolution to the deep learning segmentation network. The system uses a standard mobile phone camera input, which is then fed to a modified encoder-decoder convolutional neural network, inspired by a Fully Convolutional Dense Network that consists of a total of 11 dense blocks. A PPM is integrated into the network because of its multi-scale capability, which is useful for multi-scale tissue extraction. The shape of the tissues remains relatively constant, but the size will differ according to the severity levels. Moreover, group and shuffle convolution modules are also integrated at the decoder side of Group-PPM-Net by placing them at the starting layer of each dense block. The addition of these modules allows better correlation among the filters in each group, while the shuffle process increases channel variation that the filters can learn from. The results show that the proposed method obtains mean accuracy, mean intersection over union, Hausdorff distance, and Jaccard index performances of 0.9330, 0.8640, 11.5474, and 0.7966, respectively.

## 1. Introduction

**Figure 1:**
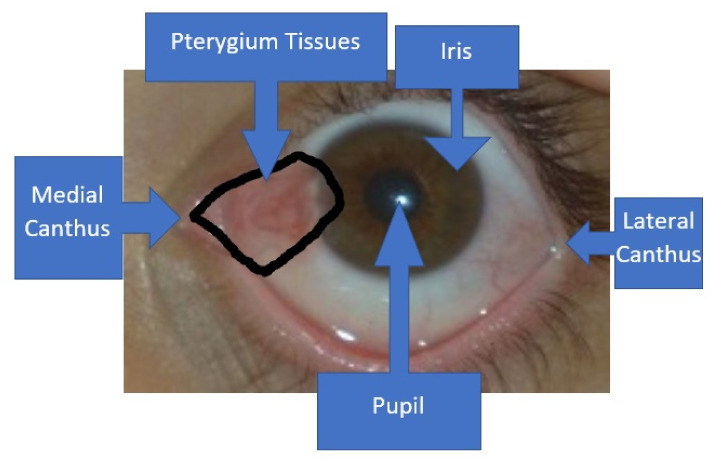
The enclosed region by the black line shows the wedge or wing shape area of the pterygium-infected tissues. The tissues will usually grow from the medial canthus region towards the pupil, as the condition becomes more severe.

Pterygium is an eye condition that is caused by the non-cancerous growth of abnormal tissues that cover the corneal regions [1]. The tissues are usually pinkish in color with a wedge shape, as shown in Figure 1. Normally, the abnormal tissues grow from the medial canthus region, rather than the lateral canthus region [2]. As the pterygium condition worsens, more abnormal tissues will encroach upon the corneal regions. Eventually, in the worst-case scenario, the patient will become blind because the tissues will block light from coming through the pupil. However, most cases will only lead to blurred vision and an uncomfortable feeling to the patients.

According to Zhou et al. [3], excessive exposure to ultraviolet (UV) radiation is usually associated with the cause of pterygium. Their findings show that radiation changes the limbal stem cells and fibroblasts, which encourages the initiation of pterygia tissues. These findings are also valid for the recurrent pterygium cases, in which the primary cause of the condition for patients that have undergone removal surgery is also highly associated with UV radiation [4]. Hence, people who live near the equator are more prone to this condition due to prolonged exposure to sunlight throughout the years. Thus, certain economic sectors, such as fishermen, construction workers, farmers, and delivery riders are the potential target groups that need to be made aware of pterygium so they can take precautionary steps to reduce their UV exposure. Moreover, knowledge of this condition will help them identify pterygium at its early stage.

A good screening system is crucial in helping healthcare practitioners to perform efficient mass screening of high-risk groups that primarily consist of low-skilled workers that happened to reside in rural areas [5]. Because most of these workers are worried about medical costs, they rarely seek periodic health advice from medical practitioners. In certain countries, such as Malaysia, volunteers typically organize health screening sessions that cover various diseases as part of community services. Due to the nature of these services, some of the volunteers are university students, and others are from the general public who do not have specialized medical skills. Therefore, an efficient screening tool will allow them to identify the risk of many diseases, including pterygium.

If the pterygium condition is detected at an early stage, the abnormal tissue encroachment into the corneal region can be halted through the continual use of eye-drops [6]. The severity of the pterygium can be assessed by determining the size of the abnormal tissues; a severe case is one in which the tissue has grown into the pupil region [7]. During the screening process, it is tedious to segment the tissues manually, especially when the number of samples is large. Therefore, an automated tool needs to be developed so that early detection of the severity level can be immediately identified. Moreover, object detection-based methods, such as YOLO V4 [8], are not pursued in this paper because of the importance of good silhouette extraction that encloses the exact region of the infected tissues to grade the severity level. A rectanglular representation of the infected tissues will be sub-optimal because two infected tissues might have the same bounding box representation, but with different sizes and shapes. Hence, this paper aims to solve this problem using a deep-learning semantic segmentation technique to identify the exact regions of the pterygium-infected tissues.

**Figure 2:**
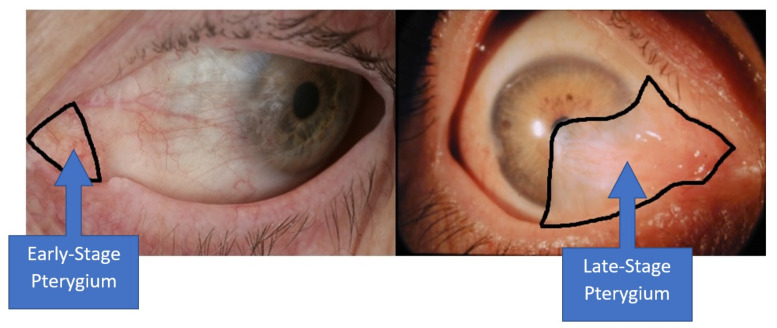
The left-hand side image shows that the pterygium is still in the early stage, where the abnormal tissue formation is limited around the medial canthus region. The right-hand side image shows that the pterygium is in the late stage, where the abnormal tissues have encroached into the pupil region.

The Fully Convolutional Dense Network (FC-DenseNet), which was designed by Jegou et al. [9], was used as the basis to develop the algorithm employed in this study. The input for the screening process is a frontal eye image that can be captured from any standard mobile phone camera. The goal of the segmentation algorithm is to come out with binary images that identify all the pterygium-infected tissues so that the severity level can be determined. However, severity grading is not the goal of this study, due to the sample size imbalance between the severity levels. In this paper, the pyramid pooling module (PPM) [10] is utilized to improve the network’s capability in handling multi-scale information, where the size of the pterygium tissues differs according to the severity level. In the early stage, the abnormal tissue encroachment will form around the medial canthus region, whereas in a severe case, the tissues will encroach to the corneal region, as shown in Figure 2. To further improve the segmentation accuracy, shuffle [11] and group [12] convolution were added to the segmentation architecture. By integrating the group convolution, segmentation features can be learned in a block way, where the filters with high correlation can be trained efficiently [13]. A shuffle operation was also added to overcome the issue of limited training variations, because only a small fraction of the input channel is utilized in standard group convolution.

Therefore, this paper utilizes a modified FC-DenseNet for the application of pterygium segmentation by embedding multi-scale capability through a pyramid pooling module and better-correlated filters through group and shuffle convolution. The utilization of dense feedforward layers allows the model to better learn the unique patterns of pterygium-infected tissues from basic to complex features. Even though more parameters have been utilized due to the usage of feedforward layers, the model is still comparatively small compared to the other state-of-the-art benchmarked model with just 13 million of trainable parameters. As a result, the proposed method has managed to achieve a processing speed of 2.63 images per second. This paper is formatted into five sections. Section 2 discusses the related works that focus on the automated pterygium screening methods and concise review on deep learning semantic segmentation. The proposed methodology, which we named as Group-PPM-Net, is explained in Section 3, and the performance results are discussed in Section 4. The conclusion and suggestions on future work are given concisely in Section 5.

## 2. Related Works

This subsection reviews some works on the automated pterygium screening and convolutional neural network-based (CNN-based) algorithms used for semantic segmentation.

### 2.1. Automated Pterygium Screening

In 2012, Mesquita and Figueiredo [14] used edge detectors to develop one of the earliest automated pterygium screening tools. They first extracted the iris region before finding the largest blob using a Sobel edge operator. The extracted region is labeled as abnormal tissues, which is then used to determine the severity level of pterygium. Another method in [15] applied a haar-like feature coupled with an AdaBoost classifier to extract pterygium-infected tissues. In [16], Fourier harmonic analysis was applied by identifying various circular diameters that can fit the infected tissue regions. A larger diameter size indicates a more severe disease. Besides that, the work in [17] analyzed the color of the pterygium-infected tissues, so that the condition can be distinguished from a cataract that has a relatively similar appearance. On the other hand, the work in [18] tried to distinguish between pterygium encroachment and astigmatism condition, which is another disease that closely resembles pterygium.

The previously discussed methods have applied simple image processing methods without using advanced supervision techniques. The work by Lopez et al. [19] adopted a convolutional neural network (CNN) to classify the eye images into a pterygium or normal class. The network is very shallow with just one layer of CNN and one dense layer with one down-pooling operator. Another compact CNN approach, which was proposed by [2], has analyzed various normalization techniques that are embedded into a network of two CNN layers and two dense layers. As shown in [20], the performance of a deep learning network can be improved using better optimization, normalization, and regularization techniques. Moreover, this method has also applied a transfer learning approach rather than using random parameter initialization. Instead of just classifying the images into two classes, the work in [1] has localized the pterygium-infected tissues by spawning various candidate boxes that might encapsulate the true infected regions. The network consists of three CNN layers and three dense layers, where the candidate boxes will be resized and tested individually so that the bounding box with the highest probability will be labeled as the infected region. The work reported in [21] semantically segmented the images by classifying each pixel into either a pterygium label or not. They modified DeepLab methods by integrating the feedforward layers into the first four CNN blocks, which are then concatenated to derive more informative feature maps.

### 2.2. Convolutional Neural Networks-Based Semantic Segmentation

The VGG family architecture [22] is a famous CNN model that specializes in the classification task; it won second place in the 2014 ImageNet Large Scale Visual Recognition Challenge (ILSVRC). Since then, the architecture has been used as the backbone in various deep learning algorithms, including object tracking [23], video summarization [24], the rehabilitation system [25], and many others. Long et al. [26] introduced the Fully Convolutional Network (FCN) in 2015, which is a semantic segmentation algorithm by deploying VGG-16 architecture as the encoder network that uses deconvolution operators to upsample the encoded image to the original input size. Three versions of FCN have been proposed that differ in the number of upsampled layers, where element-wise addition operators are utilized to combine the feedforward layers for fine-tuning the upsampled output. Another method presented in [27] also proposed a VGG-16 network as the backbone for semantic segmentation with slight modification by integrating atrous convolution. They removed the last two pooling layers and replaced the CNN striding with atrous convolution. Yu et al. [28] slightly modified the previous technique by removing both the last two pooling layers and the CNN striding operations. They introduced various dilation factors, including 2, 4, 8, and 16 atrous strides to further improve their algorithm robustness to multi-scale variations.

Instead of using a simplified architecture on the decoder side, DilatedNet [29] introduced gradual upsampling using the inverted FCN where an upsample operation is used to replace the max-pooling downsample operation. The unpooling operation uses the same localization information with regard to the corresponding max-pooling indices. The Semantic Pixel-Wise Segmentation Network (SegNet) [30] also used the same approach, where the encoder and decoder sides have the same number of convolution layers without using any pre-trained backbone model. The algorithm consisted of four CNN blocks at each of the encoder and decoder sides, where the upsample operation was done through bilinear interpolation with pooling indices to indicate the maximum location. U-Net [31], which was introduced for biomedical application has improved the segmentation network by introducing four feedforward layers that connect the encoder and decoder sides. However, no pooling indices scheme was applied, because the upsample operation is done through a transposed convolution operator. A residual layer scheme, which was popularized by the ILSVRC 2015 winner [32], has also been added to the U-Net architecture. The residual layer is applied as a skip connection that originates from the input of each down-pooling layer, which will be passed as the skip connection to the encoder side [33]. Instead of using residual connections, the work in FC-DenseNet [9] used a dense connection where the output of each CNN layer and its feedforward input are concatenated. It uses a U-Net like architecture comprised of 103 CNN layers, in which 15 layers are placed in the bottleneck section that connects the encoder and decoder sides. Contrary to a single deep network of U-Net, the work presented in [34] has stacked several shallow U-Net modules consecutively without any upsample operation.

In [10], ResNet architecture is used as the backbone where a pyramid pooling module is added to improve the multi-scale capability of the segmentation network. Several parallel CNN layers that are branched out from the same input through different pooling kernels will be combined back after performing bilinear interpolation upsample operations. The parallel branches’ role is to capture various scale information through various down-pooling operations with different kernel sizes. The work in [35] follows the same logic where parallel branches module, which they termed as atrous spatial pyramid pooling (ASPP), and is introduced by using several dilation rate factors instead of several kernel sizes of the pooling operators. Different dilation rates will capture information from different scales, where the branches will be combined back also using bilinear interpolation upsample operations. A slight improvement is proposed in [36], where the ASPP module is modified to include a normal down-pooling operation, followed by a 1 × 1 CNN layer. The decoder side has also been altered by introducing a gradual upsample process.

## 3. Methods

### 3.1. FC-DenseNet

FC-DenseNet uses a modified U-Net architecture by adding concatenated feedforward components in its dense block. Let us define a standard CNN, f(X), that takes an input layer $X_{n-1}$ as a composite function of a batch normalization layer, rectified linear unit (ReLU) activation function, convolution operator, and dropout unit.

(1) $$\begin{array}{c} X_{n}=f\left(X_{n-1}\right) \end{array}$$

**Figure 3:**
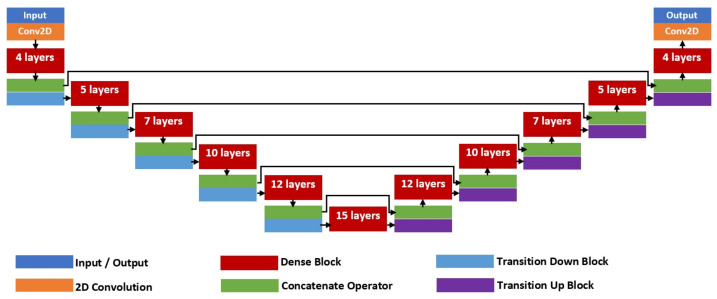
FC-DenseNet-103 architecture.

**Figure 4:**
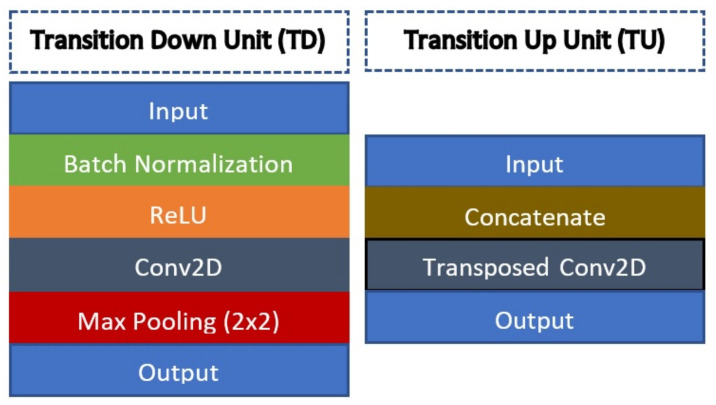
The left-hand side image shows an architecture of a single transition down (TD) unit, while the right-hand side image shows the architecture of a transition up (TU) unit.

Then, an *n*-layer output of a dense CNN block can be represented by

(2) $$\begin{array}{c} X_{n}=f\left(\left[X_{n-1},X_{n-2},\ldots ,X_{1},X_{0}\right]\right), \end{array}$$

where $X_{0}$ is the input to the respective dense block. Figure 3 shows the full architecture of FC-DenseNet-103 with a total of 103 convolution layers that comprises of five dense block units for each of the encoder and decoder networks. There is also a bottleneck unit with the smallest latent variable representation that consists of a 15-layer dense block that also connects the encoder and decoder sides. At the encoder side, a transition down (TD) unit will be applied at the end of each dense block, while at the decoder side, a transition up (TU) unit will also be applied at the end of each dense block. Since FC-DenseNet is a deep architecture, five skip connections between the encoder and decoder sides are added so that zero gradient diminishing issues can be avoided during the training process. The TD unit consists of a standard CNN composite function with an additional max-pooling operator to downsample the output feature map, as shown in the left-hand side image of Figure 4. On the other hand, a TU unit comprises of just a transpose convolution layer that takes concatenated input from the dense block.

### 3.2. Group-PPM-Net

**Figure 5:**
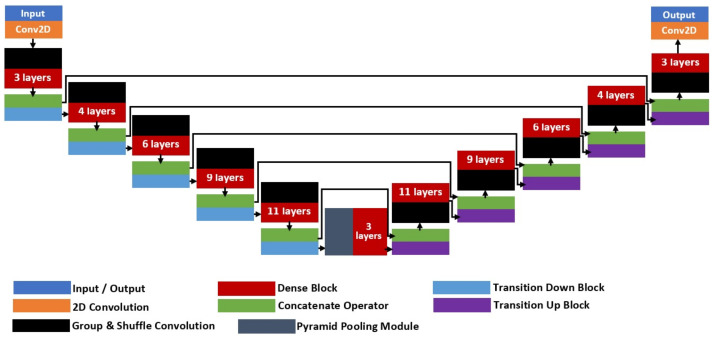
Full architecture of the proposed Group-PPM-Net for pterygium-infected tissues segmentation.

**Figure 6:**
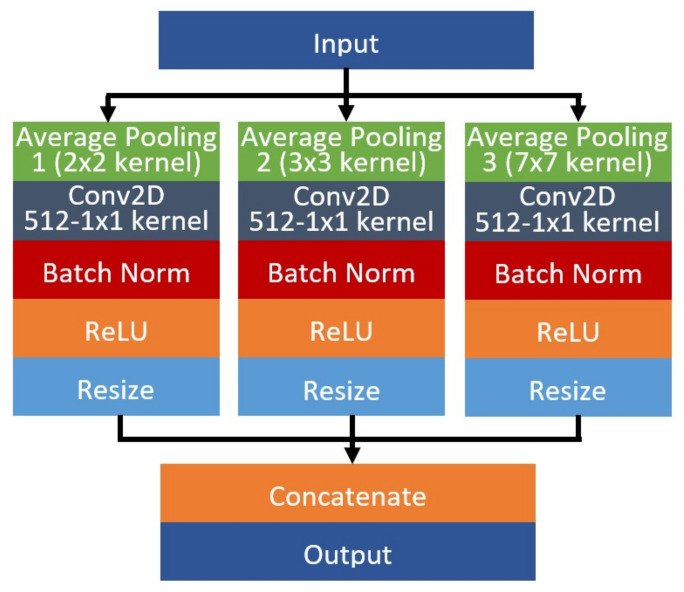
The proposed architecture of the spatial pyramid pooling module with three parallel CNN branches using pooling kernels of 2 × 2, 3 × 3 and 7 × 7.

The proposed architecture, which is termed as Group-PPM-Net, is shown in Figure 5. The architecture was inspired by the original FC-DenseNet with the integration of a spatial pyramid pooling module (PPM) and group & shuffle convolutions. The network requires a set of input data with the size of 224 × 224 pixels, where a total number of 328 images will be experimented in this study. A PPM module is added to improve the network capability in handling multi-scale cases of the pterygium-infected tissue. It has been successfully applied in many applications to improve the network capability in extracting multi-scale features, such as traffic sign recognition [37], image retrieval [38], remote sensing [39], and text detection [40]. The dataset used in this study consists of pterygium conditions that cover the early stage until the late stage. In the early stage, the size of the abnormal tissues is comparatively small compared to the pupil size, while the size is relatively big in the late stage, wherein some cases, the tissues have fully grown into the pupil region. Hence, the encroachment tissue size will differ with regard to the disease severity. One interesting point to note is that the overall shape remains relatively the same, in the form of a wedge shape, regardless of the severity level. Thus, a PPM has been added at the bottleneck layer that connects between encoder and decoder parts, followed by a three-layer dense module, so that the system is better equipped to handle multi-scale pterygium tissue detection. The placement of the PPM at the bottleneck region coincides with the smallest feature map size, which will not add too many trainable parameters to the network due to the usage of several parallel pooling layers. The proposed PPM architecture is shown in Figure 6 that consists of three parallel CNN branches, where each branch differs in the kernel pooling size, which is used to capture several scales of the encoded data. Average pooling operators are then applied with kernel sizes of 2 × 2, 3 × 3, and 7 × 7, followed by a pointwise convolution, batch normalization, and ReLU function. The resultant feature maps are then resized to the original input size before they are concatenated together.

**Figure 7:**
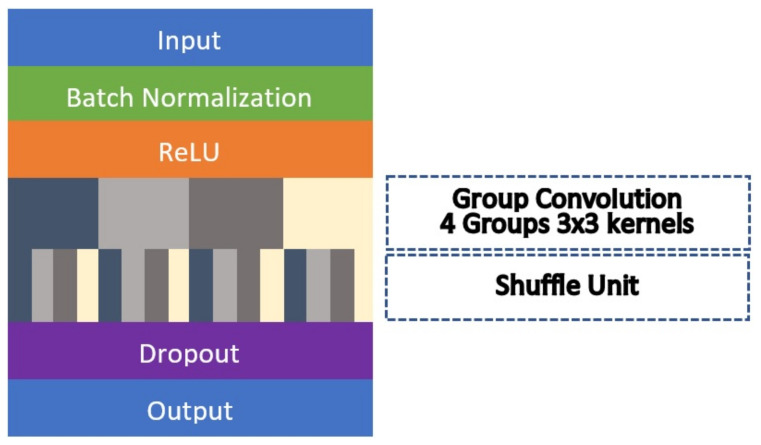
A compact architecture of a group and shuffle convolution module.

Group and shuffle convolution modules are added to further improve the segmentation accuracy. In general, group convolution allows the networks to be trained by separate sets of filters, in which the networks will not be generalized as a whole single unit. The relationship among the convolution filters are sparse in nature [41] and thus, in certain cases, the correlation among them can be improved by reducing the input channels that it can learn from. On the other hand, this approach also limits the input information that can be supplied to the filters, as it will only learn from specific channels. Hence, shuffle operation is included in our proposed architecture so that the input to the group convolutions can be diversified, where the channels will be swapped around the groups. Figure 7 shows the full network flow of the group and shuffle convolution module. Each module comprises of four groups with 3 × 3 CNN kernels. The same operations used in the dense module are carried over where the input will be normalized as a batch before ReLU activation is applied. Then, the standard CNN unit is replaced by group convolution followed by a shuffle operation. This module of group and shuffle convolution will replace the first layer of every dense block in the encoder and decoder sides. Therefore, there will be 10 modules that are applied in the whole segmentation network. However, Group-PPM-Net cannot take advantage of the multi-graphic processing unit in computing the group convolution, as the other layers in the subsequent dense blocks cannot be trained separately. The five skip layers between the encoder and decoder sides will be maintained as in the original configuration. The total number of trainable parameters of Group-PPM-Net is 13,219,138, which is less than the original FC-DenseNet with 14,594,658 trainable parameters.

## 4. Experimental Results and Discussion

### 4.1. Dataset

**Figure 8:**
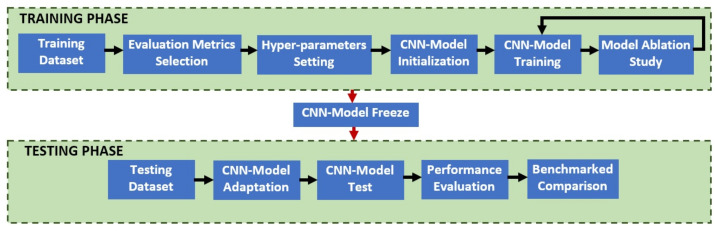
The overall workflow of the proposed approach.

The dataset used in this study was originally collected for classification purposes; hence, no segmentation ground truth images are provided. They were collected with the help of the Australian Pterygium Centre under the supervision of Professor Lawrence Hirst. Based on an exhaustive search, there is no public dataset available for the task of automated pterygium segmentation that provides the segmentation mask as the ground truth. Therefore, we built our own ground truth images using all the pterygium image cases that consist of various severity levels, ranging from the early to late stages with a total of 328 frontal eye images. The images were captured in a Joint Photographic Experts Group (JPEG) format with a high-resolution size of 4064 × 2074 pixels. Two ground truth evaluators, a biomedical researcher and a health practitioner were tasked with manually segmenting the infected tissues. The ground truth region for each image was traced using the same protocol employed in [42], which was implemented by the 2017 Automated Cardiac Diagnosis Challenge: Segmentation. First, the biomedical researcher manually traced the boundary region of the pterygium infected tissues using GNU Image Manipulation Program 2 (GIMP2) software. Then, the annotated regions were validated and corrected by the healthcare practitioner in the presence of the first evaluator. Any disagreement was discussed, and consensus on the final segmented regions was reached. GIMP2 software of version 2.10.14 was used to annotate and create the ground truth images in JPEG format with a resolution of 450 × 300 pixels. Only two output classes were used, the pixel either belonging to the pterygium tissues or not. No pre-processing method was applied to the raw images, except they were scaled to the range of [−1,1]. Similarly, no data augmentation was performed to increase the number of training data. Simple data augmentation procedures, such as translation and rotation, were not implemented in this work because of their limited effectiveness, since semantic segmentation labeling is done per pixel-wise, whereas complex synthetic data augmentation methods such as a generative adversarial network [43] were not implemented because the generated images need to be manually labeled first by healthcare practitioners before they can be part of the training data. The overall workflow of the proposed approach is shown in Figure 8.

### 4.2. Experimental Setup

The proposed Group-PPM-Net was coded in Python using a Keras front-end with a Tensorflow back-end. All the experiments were tested using an Intel i9-9900K machine that runs at a 3.60 GHz clock with a single Nvidia RTX 2080 Ti graphics card. The categorical cross-entropy loss function was applied to train the network using the Adam backpropagation method [44]. The network was trained for a minimum of 200 epochs using random initialization, where a small batch size of three images per iteration was utilized due to the limited memory storage capacity of our graphics processing unit. A fixed learning rate approach of 0.0001 was used during the network update. The algorithm was trained and validated using the dataset derived from [1]. The dataset consists of 328 frontal eye images of pterygium patients, which are split randomly according to the ratio of 1:3 for testing and training purposes. As seen in Figure 8, the proposed method and all the benchmarked algorithms were trained until convergence, where the training accuracies have converged to the optimal value of 1, except for the Pyramid Scene Parsing Network (PSP-Net), which converged to 0.85. The convergence speed varies between the algorithms with DeepLab V3+ achieving the fastest convergence, while FCN started slowly and converged rapidly after 32 epochs. The training and testing performances (Table 1) prove that the issues of over-fitting and under-fitting due to the limited number of training data are minimal in this case.

### 4.3. Performance Metrics

Four standard segmentation metrics are used to evaluate the proposed method performance, which are pixel-wise mean accuracy ($\bar{Acc}$, class-based mean intersection over union ($\bar{IoU}$), the Hausdorff distance ($H_{dist}$), and Jaccard index ($J_{index}$). Let $I_{i}$ represents a pixel at location *i* with the total number of pixels $T_{p}$, then $L_{i,gt}$ is its ground-truth label, while ${\hat{L}}_{i}$ is the label predicted by the network. Since this work only considers a binary problem, the class α is either 0 (non-pterygium) or 1 (pterygium). Note that the logical and is denoted by ∧ and the logical or operator is denoted by ∨, and hence $\bar{IoU}$ and $\bar{Acc}$ are the following:

(3) $$\begin{array}{cc} \bar{IoU} & =\frac{\sum_{\forall i}\left(L_{i,gt}==\alpha \wedge {\hat{L}}_{i}==\alpha \right)}{\sum_{\forall i}\left(L_{i,gt}==\alpha \vee {\hat{L}}_{i}==\alpha \right)} \end{array}$$

(4) $$\begin{array}{cc} \bar{Acc} & =\frac{\sum_{\forall i}\left(L_{i,gt}==\alpha \wedge {\hat{L}}_{i}==\alpha \right)}{T_{p}}. \end{array}$$

For $H_{dist}$ and $J_{index}$, only the segmented pterygium region is concerned. Let *M* and *N* be the two points set that represent the segmented regions of the ground truth ($R_{M}$) and network prediction ($R_{N}$), respectively. A one-directional $H_{dist}$ can be written as:

(5) $$\begin{array}{c} H_{dist}^{single}\left(M,N\right)=\max_{m\in M}\left\{\underset{n\in N}{\sup}||m-{n||}_{2}\right\}. \end{array}$$

Hence, a bi-directional $H_{dist}$ can be formulated as follows:

(6) $$\begin{array}{c} H_{dist}\left(M,N\right)=\max\left\{H_{dist}^{single}\left(M,N\right),H_{dist}^{single}\left(N,M\right)\right\}. \end{array}$$

For $J_{index}$, the formula can be written as

(7) $$\begin{array}{c} J_{index}\left(M,N\right)=\frac{|R_{M}\cap R_{N}|}{|R_{M}\cap R_{N}|}. \end{array}$$

### 4.4. Performance Benchmark with the State-of-the-Art CNN Segmentation Models

**Figure 9:**
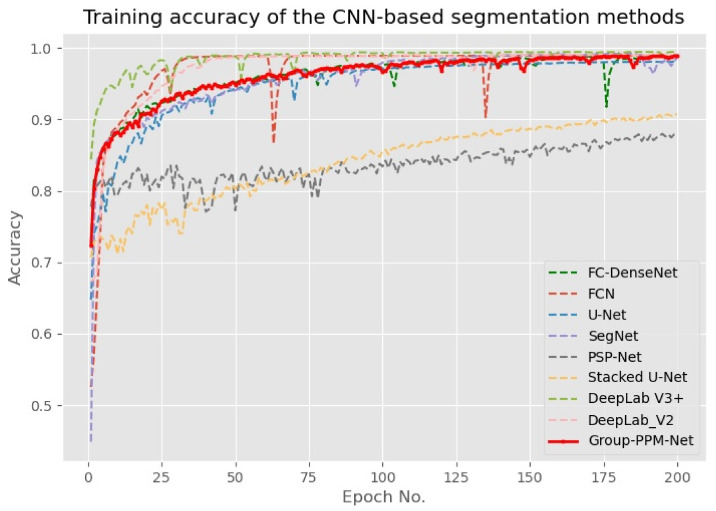
Training graph of the segmentation methods that have been trained for 200 epochs using an Adam optimizer with a fixed learning rate of 0.0001.

To compare the state-of-the-art performance of the methods, eight other CNN-based semantic segmentation models were tested, including FCN [26], SegNet [30], U-Net [31], stacked U-Net [34], FC-DenseNet [9], PSP-Net [10], DeepLab V2 [35], and DeepLab V3+ [36]. All the methods were trained using the same setup without any pre-trained parameters, but the input size to the networks remained the same as in the original design. Table 1 shows the performance of the proposed method and the state-of-the-art benchmarked methods, while Table 2 shows the ablation study that measures the segmentation performance after the addition of each component to the proposed method. The only pre-processing step that was applied is image normalization that maps the input images to the range of [−1,1], and this step was applied to all the benchmarked methods. Table 1 shows the performance results of the proposed Group-PPM-Net and the selected bench-marked methods. The best pixel-wise mean accuracy was returned by the Group-PPM-Net with 0.9349, which is relatively higher than the second-best $\bar{Acc}$ produced by SegNet with 0.9185. However, the worst $\bar{Acc}$ of just 0.7683 was returned by DeepLab V3+. This low-performance value can be attributed to the over-fitting problem, as proven by the training graph shown in Figure 9. DeepLab V3+ achieved the fastest convergence state, which is close to 1.0, just after 50 epochs of training, but its accuracy was not good during testing. Interestingly, the training accuracy for PSP-Net does not converge to 1.0 after 200 epochs like it does for the other methods, but its $\bar{Acc}$ of 0.7824 is close to the training accuracy of around 0.85. Hence, it produces the lowest performance drop between the training and testing dataset, which can be attributed to its robustness.

**Figure 10:**
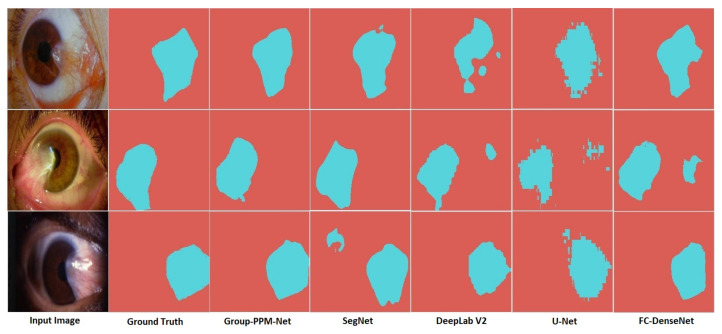
Output samples of the segmented pterygium-infected tissues.

Group-PPM-Net also returned the best $\bar{IoU}$ of 0.8669, followed by SegNet and DeepLab V2 with $\bar{IoU}$ of 0.8354 and 0.8327, respectively. A 0.3 factor improvement in the mean IoU value has a significant impact in determining the severity level. These small differences can be very challenging when attempting to determine the severity level, as shown by the output samples in Figure 10. The samples show that the other bench-marked methods tend to produce a false detection that will affect the size of the extracted pterygium-infected tissues, which directly affects the diagnosis of the pterygium severity level. Moreover, U-Net produces a significant amount of jagged segmentation outputs, which make the boundaries look uneven, which also affects the severity level assessment. It is important to note that the U-Net output in the second row cannot correctly segment the tissues that have encroached into the pupil region. This miss-detection will decrease the quality of the severity level assessment. Hence, a small increment in $\bar{IoU}$ is important for the pterygium segmentation as it directly correlates with the accuracy of the disease assessment. Besides that, Group-PPM-Net produced the best $\bar{IoU}$ with a relatively light-weight model of just 13,219,138 trainable parameters. That result is even smaller than the original FC-DenseNet of 14,594,658 trainable parameters due to the smaller kernel size used in the group convolutions.

In terms of $H_{dist}$ and $J_{index}$, Group-PPM-Net still produced the best results with the lowest Hausdorff distance with just 11.9989 pixels, followed by the original FC-DenseNet and U-Net with 13.2491 and 13.9372 pixels, respectively. Although DeepLab V2 produced good $\bar{Acc}$ and $\bar{IoU}$, its $H_{dist}$ is relatively high compared to the other methods. This performance fluctuation is caused by the wrongly segmented region in a few cases where the size of the detected region differs significantly from the ground truth. This reasoning is also supported by the result of its Jaccard index, where DeepLab V2 also produces a relatively low segmentation performance with $J_{index}=0.7158$. Similarly, the Group-PPM-Net and FC-DenseNet returned the two best $J_{index}$ of 0.7946 and 0.7512, respectively. The results also showed that the addition of the PPM and Group & Shuffle modules significantly increased the original network performance. For networks that use a symmetric encoder and decoder configuration, SegNet and U-Net performed relatively well for both measures of $H_{dist}$ and $J_{index}$. This can be attributed to the fact that they both have a better up-sampling approach than the FCN and DeepLab V2. Besides that, U-Net is the fastest algorithm that can be processed at 4.0951 images per second, even though it uses more than 31 million parameters. Its architecture is straightforward with just repeated downsampled and upsampled convolution operators with few skip connections. On the other hand, the stacked U-Net with just 3 million parameters also requires approximately the same processing time, although it has a lightweight network design. This is because it is composed of a much deeper network architecture than the U-Net, where it uses a small number of parameters for each layer of cascading encoder-decoder units. Besides that, Group-PPM-Net is found to be the second slowest algorithm, where only 2.6295 images can be processed per second. The main processing burden can be attributed to several group and shuffled operations, where the computational burden is high even though the number of parameters does not increase that much. Finally, FCN was found to be the slowest network because of its large filter sizes, as proven by its high memory requirement with more than 134 million parameters.

### 4.5. Ablation Study of the Group-PPM-Net

Table 2 shows the performance results of the Group-PPM-Net ablation study. Two modifications were suggested to improve the original FC-DenseNet through the introduction of PPM at the bottleneck layer and replacing the first layer in the dense block with the group and shuffle convolution module. According to Table 2, the addition of the individual component of either the group or shuffle operator resulted in worse segmentation performance compared to the original FC-DenseNet, as measured by all performance metrics. This is because group and shuffle operators perform the best if they are combined, in which a shuffle operator allows the group convolution to derive its input from the other group channels. Contrary to that, the addition of a single PPM module to FC-DenseNet managed to slightly improve the segmentation performance, as measured by all performance metrics, albeit being slightly slower to process. When two modules were combined to the original FC-DenseNet, the segmentation performance follows the same trend as in the single module addition, where the algorithm’s performance was worse. When the original FC-DenseNet was applied together with both group & shuffle modules, the segmentation performances increased in terms of $\bar{Acc}$ and $\bar{IoU}$ with 0.9186 and 0.8348, respectively. However, its general performance was slightly degraded when it was measured through $H_{dist}$ and $J_{index}$, where $H_{dist}$ reduced to 14.1382 pixels and $J_{index}$ reduced to 0.7368 pixels. These results show that the combined group and shuffle modules managed to improve network capability in distinguishing the true negative, while reducing the true positive detection.

Therefore, the three modules were combined together to further improve the segmentation performance to produce a significant segmentation improvement. The resultant Group-PPM-Net is just slightly slower than the original FC-DenseNet, with 2.6108 images per second compared to 2.7242 images per second. A further test was also performed to measure the algorithm performance with regard to the placement of group & shuffle modules. As a reference, the original Group-PPM-Net applies the group & shuffle modules at the encoder and decoder sides of the network. The results in Table 2 indicate that the best performance was obtained when the group & shuffle modules were only applied at the encoder side, where its $\bar{Acc}$, $\bar{IoU}$ and $J_{index}$ increased to 0.9330, 0.8640 and 0.7966, respectively. Contrary to this, group & shuffle modules will become less effective when it is applied at the decoder side only, where only $H_{dist}$ improved to 10.3480. Therefore, the best variant of Group-PPM-Net was obtained when group & shuffle modules were applied to the encoder side only. The main reasoning behind this lower performance can be attributed to the nature of the network up-sample operation. During the down-sampling process, the network goal is to encode the information into a smaller set of latent variables, which is inlined with the goal of a group & shuffle modules addition, which is to better extract the unique features. During the up-sampling process, the addition of the group & shuffle modules will dilute the reconstructed features, as the channel will be shuffled according to its group. There is no benefit in shuffling the features at this stage, because it will create more feature randomness, which is in contrast with the goal of feature reconstruction. However, the performance difference was small among all three variants of the Group-PPM-Net, which performed better than the benchmarked state-of-the-art methods.

## 5. Conclusions and Future Works

In conclusion, Group-PPM-Net was successfully developed and validated for pterygium-infected tissue segmentation. Two innovative modules, PPM and group convolution, were explored to modify the original FC-DenseNet for better segmentation accuracy. PPM was added because of its multi-scale capability, which is useful for detecting pterygium from the early stage to the late stage, since the infected tissues have a relatively similar shape but different encroachment size. The group and shuffle convolution modules were also integrated to better train the network, where the best performance was obtained if they were placed at the encoder side of the Group-PPM-Net. The best variant of the Group-PPM-Net obtained segmentation performances of $\bar{Acc}$, $\bar{IoU}$, $H_{dist}$, and $J_{index}$ of 0.9330, 0.8640, 11.5474 and 0.7966, respectively. In future works, atrous and separable convolutions will be explored to further improve the segmentation accuracy and to reduce the computational burden of the networks.

## Figures and Tables

**Table 1:** Performance results of the Group-PPM-Net and the benchmarked methods in segmenting the pterygium-infected tissues.

Method	$\bar{\mathrm{Acc}}$	$\bar{\mathrm{IoU}}$	$H_{\mathrm{dist}}$	$J_{\mathrm{index}}$	Image/Second	Parameters
DeepLab V3+ [36]	0.7683	0.5575	64.6621	0.2077	2.4778	41,051,088
Stacked U-Net [34]	0.8046	0.6420	41.4411	0.608	3.7186	3,035,650
PSP-Net [10]	0.8884	0.7824	35.1803	0.6882	2.3976	27,838,400
FCN [26]	0.9047	0.8110	15.2212	0.6909	2.5622	134,393,428
FC-DenseNet [9]	0.9117	0.8239	13.2491	0.7512	2.7242	14,594,658
U-Net [31]	0.9128	0.8251	13.9372	0.7255	4.0951	31,032,834
DeepLab V2 [35]	0.9169	0.8327	22.5102	0.7158	2.5927	71,419,720
SegNet [30]	0.9185	0.8354	14.6579	0.7386	3.9844	29,444,166
Group-PPM-Net	0.9329	0.8632	11.9989	0.7946	2.6295	13,219,138

**Table 2:** Performance results of the Group-PPM-Net ablation study.

Method	$\bar{\mathrm{Acc}}$	$\bar{\mathrm{IoU}}$	$H_{\mathrm{dist}}$	$J_{\mathrm{index}}$	Image/Second
FC-DenseNet	0.9117	0.8239	13.2491	0.7512	2.7242
FC-DenseNet + Group	0.8623	0.7508	18.3827	0.6826	2.7023
FC-DenseNet + Shuffle	0.6774	0.7269	27.2436	0.6294	2.6866
FC-DenseNet + PPM	0.9190	0.8402	11.4322	0.7795	2.6215
FC-DenseNet + Group + PPM	0.8504	0.7324	19.2126	0.669	2.6844
FC-DenseNet + Shuffle + PPM	0.9099	0.8243	14.7687	0.7556	2.6789
FC-DenseNet + Group + Shuffle	0.9186	0.8348	14.1382	0.7368	2.6635
Group-PPM-Net (Encoder)	0.9330	0.8640	11.5474	0.7966	2.6108
Group-PPM-Net (Decoder)	0.9327	0.8626	10.3480	0.7949	2.6269
Group-PPM-Net (Both sides)	0.9329	0.8632	11.9989	0.7946	2.5823

## Abbreviations

The following abbreviations are used in this manuscript:

PPM	Pyramid Pooling Module
CNN	Convolutional Neural Networks
ILSVRC	ImageNet Large Scale Visual Recognition Challenge
ASPP	Atrous Spatial Pyramid Pooling
ReLU	Rectified Linear Unit
GIMP2	GNU Image Manipulation Program 2
FC-DenseNet	Fully Convolutional Dense Network
FCN	Fully Convolutional Network
PSP-Net	Pyramid Scene Parsing Network
SegNet	Semantic Pixel-Wise Segmentation Network
TD	Transition Down
TU	Transition Up
